# Circulating Biomarkers are Associated with Impaired Right Ventricular Circumferential and Longitudinal Strain Measured by Cardiac Magnetic Resonance Imaging in Children and Adolescents with Repaired Tetralogy of Fallot

**DOI:** 10.1007/s00246-025-04035-9

**Published:** 2025-09-23

**Authors:** Chandni Patel, Andrea L. Jones, Angeli Thomas, Michael DiLorenzo, Antara Mondal, Alexis Z. Tomlinson, Danish Vaiyani, Mark Fogel, Elizabeth Goldmuntz, Laura Mercer-Rosa

**Affiliations:** 1 Division of Cardiology, The Children’s Hospital of Philadelphia, 254 Easton Ave, MOB2, New Brunswick, NJ 08901 USA; 2 The Children’s Hospital of Philadelphia, Philadelphia College of Osteopathic Medicine, Philadelphia, PA USA; 3 Division of Cardiology, Columbia University Irving Center/Morgan Stanley Children’s Hospital, New York, NY USA; 4 Department of Biomedical and Health Informatics, The Children’s Hospital of Philadelphia, Philadelphia, PA USA

**Keywords:** Tetralogy of Fallot, CMR strain imaging, Biomarkers, RV remodeling, Ventricular deformation

## Abstract

Prior studies have shown that circulating biomarkers are associated with increased myocardial fibrosis and worse outcomes in patients with surgically repaired tetralogy of Fallot (rTOF). However, associations of circulating biomarkers with right and left ventricular myocardial deformation using strain by cardiac magnetic resonance (CMR) imaging remain understudied. We aimed to investigate whether these associations in rTOF youth undergoing CMRs inform ventricular remodeling. Eight circulating biomarkers were measured in rTOF patients at the time of CMR. Right and left ventricular (RV, LV) longitudinal (GLS) and circumferential (GCS) strain were measured offline using cvi42 Tissue Tracking®. Associations between strain and CMR RV/LV parameters and biomarkers were assessed using correlation coefficients and linear regression models adjusted for age at CMR and sex. Fifty-one patients were analyzed (51% female). Worse RVGLS was associated with worse RVEF (ejection fraction), LVEF and LVGS. RVGCS was associated with RVEF. Higher MMP1 was associated with worse RV remodeling, including increased RV end-diastolic volume, lower RVEF and worse RVGCS. Higher sST2 was associated with worse LVEF. Higher NTproBNP was associated with worse RVGCS, more notable in females. We identified three biomarkers (MMP1, sST2 and NTproBNP) associated with ventricular remodeling. Examining changes in these biomarkers over time and their association with ventricular remodeling will help determine if circulating biomarkers can identify early ventricular dysfunction in rTOF. RVGCS may be an important measure to study further in the assessment of RV function after TOF repair.

## Introduction

Despite improvements in long-term prognosis and survival, patients with tetralogy of Fallot (TOF) continue to face late consequences of surgical repair. These are particularly related to the deleterious effects of residual volume and/or presssure loading of the right ventricle (RV), resulting in maladaptive RV remodeling [1–4].

Myocardial fibrosis occurs during remodeling of the RV under stress [5]. It has been identified histologically prior to surgical repair for TOF [6, 7]. Following TOF repair, there is evidence of altered extracellular matrix (ECM) in patients undergoing pulmonary valve replacement (PVR) [8]. Greater extent of diffuse myocardial fibrosis as measured by cardiac magnetic resonance imaging (CMR) is associated with adverse outcomes such as cardiac arrest, ventricular tachycardia, heart failure and death [2, 6, 9, 10].

In addition to measuring fibrosis and standard parameters including volume, mass and flows, CMR allows for the measurement of myocardial deformation (strain) [11, 12]. Strain imaging has the potential to detect myocardial injury more reliably and earlier than conventional measures of cardiac function (such as ejection fraction) and thereby, potentially allows for timelier intervention [13, 14]. Impaired RV and left ventricle (LV) strain parameters by CMR have been associated with diffuse fibrosis as well as adverse clinical outcomes in TOF, such as ventricular tachycardia or death [15, 16].

We had previously identified a panel of circulating biomarkers associated with myocardial processes including fibrosis, ECM turnover, myocardial stretch, collagen synthesis, and inflammation. In a previous study, we had evaluated the association of serum biomarkers with traditional volumetric and functional parameters of CMR [17]. In this translational cross-sectional study, we first assessed the associations between traditional RV/LV CMR measures and CMR strain measures. We then sought to identify cross-sectional associations between biomarkers and ventricular function using longitudinal and circumferential strain by CMR in youth with repaired tetralogy of Fallot (rTOF). Identifying correlations between circulating biomarkers of myocardial fibrosis and ventricular function could inform early RV dysfunction, which remains challenging to identify in this patient population.

## Methods

We conducted a single-center cross-sectional study of patients with rTOF presenting for clinically indicated CMR. The study cohort was previously described [17]. Of the sixty patients analyzed in our previous study, nine patients were excluded in our current study due to inadequate image quality for strain analysis. This study was approved by the Institutional Review Board at the Children’s Hospital of Philadelphia. Blood samples were collected from TOF patients at the time of standard of care CMR at our institution between October 2015 and September 2017 [17]. Galectin-3 (Gal-3), microRNA 21 (miR21), matrix metalloproteinases 1 and 9 (MMP1 and MMP9, respectively), carboxy-terminal propeptide of procollagen type 1 (PICP), amino-terminal propeptide of procollagen type 3 (PIIINP), soluble serum stimulation-2 (sST2) and N-terminal-pro hormone brain natriuretic peptide (NTproBNP) were measured using assays as previously described [17]. We chose Gal-3 as it is implicated in promoting collagen formation and thereby modulating myocardial fibrosis [18, 19]. Similarly, miR21 is thought to be associated with cardiac hypertrophy and fibrosis as a TGF-beta activator [17, 20]. MMP1 and MMP9 are associated with collagen turnover [17, 21]. PICP and PIIINP are associated with collagen synthesis [21, 22]. sST2, which was previously called “suppressor of tumorgenicity 2”, functions as a decoy receptor, binding interleukin 33 (IL-33) thus blocking the IL-33/ST2 ligand interaction, acting as a regulator of IL-33 activity. It has been implicated in fibrosis, tissue inflammation and damage, inflammation and remodeling. sST2 is primarily involved in inflammation, but has been shown to be upregulated in cardiac myocytes under strain and to have cardioprotective effects [23]. NTproBNP is a widely used biomarker because it is produced in situations of myocardial stretch from pressure and volume overload on the heart [17, 24, 25].

### CMR Strain Measurements

CMRs were performed on 1.5 Tesla Siemens Avanto FIT MRI scanners (Siemens Healthineers, Erlangen, Germany). Details on the CMR protocol used at our center and for the measurement of right ventricular end-systolic (RVESV) and end-diastolic volume indexed to BSA (RVEDV), indexed mass, ventricular function, and pulmonary regurgitant fraction, have been previously published [17, 26]. Exclusion factors included suboptimal CMR image quality or an incomplete study.

Steady state free precession cine images were analyzed by a single observer using cvi42 Tissue Tracking® (Circle Cardiovascular Imaging Inc., Alberta, Canada) software. We measured LV and RV longitudinal and circumferential strain as these are the parameters most widely used to assess RV and LV function [14]. The endocardial and epicardial borders were manually delineated in the short axis stack and cine 4-chamber views, and, when available, were delineated in the LV 2-chamber, RV 2-chamber, and 3-chamber (LVOT) views. The RV 2-chamber view was not available for analysis in 3 patients; the LV 2-chamber view was not available in 6 patients; and the LVOT view was not available in 5 patients. The automated tracking software in cvi42 was then used to calculate strain. RV longitudinal strain was derived from 4-chamber images and 2-chamber images. LV longitudinal strain was obtained from the 4-chamber, 3-chamber and 2-chamber views. Circumferential strain was analyzed from the short axis images. RV and LV global longitudinal and RV and LV global circumferential strain were recorded.

Strain measurements were repeated in 9 patients to assess for intra-observer reliability using intraclass correlation coefficients (ICC). Measurements were considered poorly reliable if ICC < 0.50, moderately reliable if ICC values were between 0.5 and 0.75. Good reliability was defined as ICC values between 0.75 and 0.9, and ICC values greater than 0.90 indicated excellent reliability. Intra-observer correlation was good to excellent for the strain measurements by CMR: LV GLS (ICC 0.90 [0.76, 1.03]), LV GCS (ICC 0.98 [0.95, 1.01]), RV GLS (ICC 0.86 [0.68,1.04]), and RV GCS (ICC 0.94 [0.86, 1.02]).

### Statistical Analysis

Patient demographics and surgical characteristics were collected from the electronic medical record. Categorical variables are presented as frequency (N) and percentage (%). Continuous variables are presented as median (25th, 75th percentile) or mean (standard deviation). Demographic characteristics are presented for the overall sample and by groups according to RV dilation. Historically, thresholds for RV dilation in asymptomatic rTOF patients under consideration for PVR has been defined as RVEDV greater than 150–160 mL/m^2^ [27–29]. Thus, in our study, we used a parameter of RVEDV greater than or equal to 160 mL/m^2^ to ensure a cohort with evident RV dilation. Subgroup demographics were compared using Fisher’s exact test (categorical variables) or Wilcoxon rank sum test (continuous variables).

We obtained correlation coefficients within CMR parameters to explore the association of volumes and volume-based ventricular function (EF) with type of myocardial deformation (longitudinal or circumferential strain) [30].

### Biomarkers

The following biomarkers were measured in serum for the purposes of the study: Gal3, miR21, MMP1, MMP9, PICP, PIIINP, ST2, and NTproBNP. All biomarkers were log-transformed to adjust for the skewness in the distributions, except for PIIINP which was dichotomized into groups for PIIINP <  = 20 ng/mL and PIIINP = 20 ng/mL because many patients had values greater than 20 ng/mL, the upper limit of detection.

### Model Details

Linear regression was performed for all biomarkers, except PIIINP where logistic regression was performed. Univariable regression was first performed to determine the association between each biomarker (outcome) and each strain parameter or ejection fraction (EF) (exposure). For univariable associations with *p*-value < 0.10, multivariable regression was performed adjusting for age at time of CMR and sex along with the significant strain or EF covariate. For NTproBNP, the additional variable of RVEDV was also included. Multivariable model *p* values < 0.05 were considered significant. All analyses were performed using R Statistical Software (version 4.2.3; R Core Team 2021) [31].

## Results

### Patient and Clinical Characteristics

Fifty-one patients with TOF who underwent clinically indicated CMR also had blood samples collected for biomarker measurements and were included in this study. There were 25 (49%) males and 26 (51%) females. The majority of the patients were White (88%). The median age at the time of initial TOF surgery was 65 days [IQR: 14,172]. Most patients underwent a repair using a transannular patch (90%) with others having an RV-PA conduit (5.9%), or valve sparing repair (3.9%). Six patients had Blalock-Taussig-Thomas (BTT) shunt palliation prior to complete repair. Twelve patients (24%) had a history of pulmonary valve replacement at the time of CMR. The median age at time of CMR was 15 years [10, 22]. Baseline characteristics are listed in Table 1.

**Table 1:** Patient, CMR and biomarker characteristics

Characteristic	Overall, *N* = 51^a^	RV EDV < 160 mL/m^2^, *N* = 43^a^	RV EDV > = 160 mL/m^2^, *N* = 8^a^	*p*-value^b^
Age at CMR (years)	15 (10, 22)	15 (10, 22)	15 (10, 19)	0.7
Race				0.2
White	45 (88%)	39 (91%)	6 (75%)	
Black	1 (2.0%)	1 (2.3%)	0 (0%)	
Asian	3 (5.9%)	1 (2.3%)	2 (25%)	
Other	2 (3.9%)	2 (4.7%)	0 (0%)	
Age at surgery (days)	65 (15, 168)	62 (11, 193)	109 (59, 118)	> 0.9
BTTS* pre-rTOF	6 (12%)	6 (14%)	0 (0%)	0.6
TOF repair type if known:				> 0.9
Transannular Patch	45 (88%)	37 (86%)	8 (100%)	
RV-PA conduit	3 (5.9%)	3 (7.0%)	0 (0%)	
Valve sparing	2 (3.9%)	2 (4.7%)	0 (0%)	
History of PVR**	12 (24%)	7 (16%)	5 (63%)	0.012
Age at PVR (years)	16.9 ± 7.2	18.8 ± 8.7	14.6 ± 4.6	0.36
Sex				0.024
Female	26 (51%)	25 (58%)	1 (13%)	
Male	25 (49%)	18 (42%)	7 (88%)	
**CMR parameters**				
LV EDV	66 (58, 77)	65 (57, 71)	81 (72, 84)	0.002
LV ESV	23 (20, 27)	21 (19, 26)	28 (27, 33)	0.003
LV stroke volume	43 (38, 47)	42 (36, 46)	50 (45, 53)	0.017
LV EF	64.0 (60.0, 69.0)	65.0 (60.5, 69.5)	62.0 (59.2, 64.8)	0.2
LV cardiac output	3.20 (2.90, 3.70)	3.20 (2.85, 3.70)	3.21 (2.98, 3.53)	0.8
RV EDV	114 (100, 144)	112 (94, 126)	170 (167, 181)	< 0.001
RV ESV	51 (41, 64)	50 (39, 55)	82 (74, 91)	< 0.001
RV stroke volume	68 (56, 81)	64 (54, 74)	92 (87, 96)	< 0.001
RV EF	58.0 (54.0, 61.1)	58.0 (55.0, 62.0)	52.5 (47.8, 56.0)	0.028
RV cardiac output	5.00 (4.22, 6.19)	4.96 (4.00, 5.75)	6.05 (5.53, 7.43)	0.016
Pulmonary RF%*	38 (26, 43)	35 (23, 43)	44 (37, 48)	0.076
LV GCS	−19.60 (−21.40, −16.90)	−19.70 (−21.75, −17.35)	−17.40 (−20.68, −16.68)	0.2
LV GLS	−18.50 (−20.30, −17.00)	−18.70 (−20.55, −17.10)	−18.20 (−18.60, −16.03)	0.2
RV GLS	−17.3 (−20.1, −15.3)	−17.8 (−20.3, −15.6)	−16.4 (−17.0, −15.1)	0.2
RV GCS	−17.87 (−19.67, −15.50)	−17.87 (−19.85, −15.75)	−17.61 (−19.08, −15.16)	0.5
Viability Defects	32 (63%)	27 (63%)	5 (63%)	> 0.9
**Biomarkers**				
Gal3	5.39 (4.60, 5.86)	5.36 (4.58, 5.93)	5.48 (4.94, 5.68)	> 0.9
miR21	28.53 (27.58, 29.08)	28.64 (27.54, 29.15)	28.37 (27.67, 28.66)	0.6
MMP1	15 (11, 30)	14 (10, 25)	32 (20, 41)	0.033
MMP9	129 (85, 211)	129 (89, 226)	117 (65, 153)	0.3
PICP	1,199 (838, 1777)	1222 (838, 1777)	1185 (1065, 1770)	> 0.9
PIIINP	20.00 (17.89, 20.00)	20.00 (16.81, 20.00)	20.00 (20.00, 20.00)	0.068
sST2	15 (12, 20)	15 (11, 20)	17 (14, 20)	0.4
NTproBNP	0.30 (0.10, 0.73)	0.30 (0.11, 0.62)	0.26 (0.08, 1.15)	0.9

^a^*n* (%); Median (IQR) or Mean (± SD)

^b^Fisher’s exact test; Wilcoxon rank sum test; Wilcoxon rank sum exact test

*BTTS: Blalock-Taussig-Thomas Shunt; **PVR: pulmonary valve replacement; *^RF: pulmonary regurgitant fraction%

*LV* left ventricle, *RV* right ventricle, *ESV* end-systolic volume, *EDV* end-diastolic volume, *EF* ejection fraction, *GLS* global longitudinal shortening, *GCS* global circumferential shortening

### Comparison by Severity of RV Dilation

Eight patients were in the greater RV dilation group. The group with greater RV dilation had a greater proportion of males and greater LV dilation, measured both as LVESV and LVEDV. This group had greater RVESV with corresponding greater RV stroke volume, but lower RVEF. Further, this group had greater severity of pulmonary regurgitation, although this was not statistically significant. There was a greater proportion of RV dilation within the 12 patients that had previously undergone a PVR compared to the whole cohort (5 out of 12). RV and LV strain were comparable between RV dilation-severity groups. MMP1 was higher in the more dilated group, and other biomarkers were comparable (Table 1).

### CMR Correlations with RV Strain

**Fig. 1:**
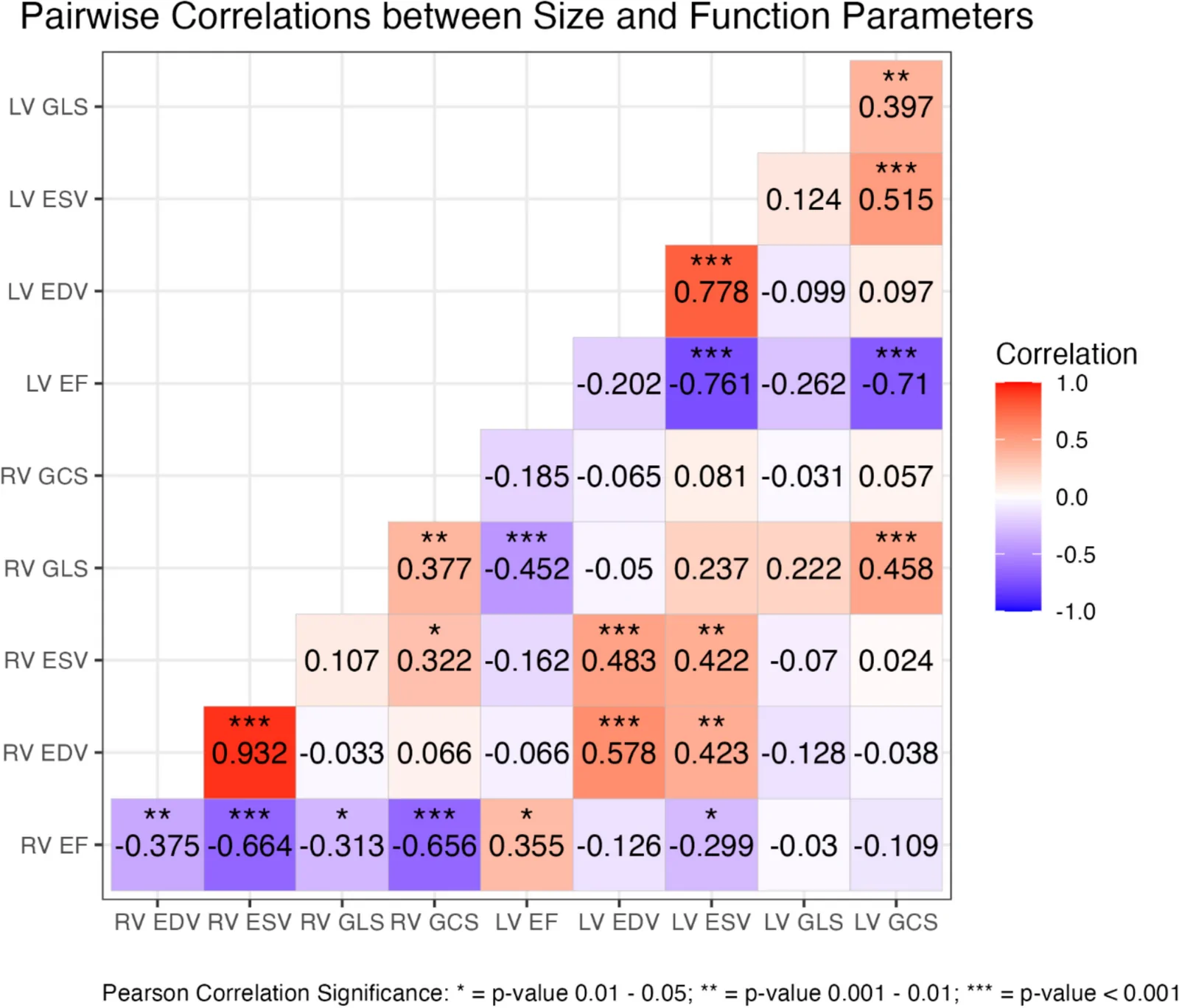
Correlation matrix of ventricular volumes and strain: *LV* left ventricle, *RV* right ventricle, *ESV* end-systolic volume, *EDV* end-diastolic volume, *EF* ejection fraction, *GLS* global longitudinal shortening, *GCS* global circumferential shortening

LVEF and LVGCS were worse in patients with worse RVGLS (Fig. 1). Lower RVEF was seen in patients with worse RVGLS, though RVGLS was not correlated with the individual volumetric parameters (RVEDV and RVESV) used in the RVEF calculation. Lower RVSV and RVEF were seen in patients with worse RVGCS, and worse RVGCS was moderately correlated with worse RVEF (correlation coefficient −0.66, *p* < 0.001) (Fig. 1).

#### Adjusted Models of Biomarkers on Ventricular Ejection Fraction and Strain

Univariable regression models demonstrated three of the biomarkers (MMP1, sST2, NTproBNP) to have significant associations with function and strain parameters (Table 2). Multivariable regression models showed that higher MMP1 levels were associated with worse RVGCS and RVEF, independent of age at CMR and sex (Table 3). Greater sST2 levels were associated with worse LVEF, and higher levels of NTproBNP levels were associated with worse RVGCS, adjusting for age at CMR and sex. NTproBNP levels were lower in male patients. The association of NTproBNP with RVGCS was not affected when RVEDV was added to the model. No other significant associations were found between MMP9, Gal3, PICP, miR21 and PIIINP and strain parameters.

**Table 2:** Univariable linear regression model of statistically significant biomarkers and ventricular size and functional parameters

Outcome	Characteristic	Beta	95% CI	*p*-value
MMP1	RV GCS	0.07	0.01, 0.14	0.034
	RV GLS	0.06	0.00, 0.12	0.059
	LV GCS	0.05	−0.03, 0.12	0.24
	LV GLS	0.05	−0.03, 0.13	0.23
	RV EF	−0.04	−0.07, −0.01	0.005
	LV EF	−0.01	−0.05, 0.02	0.39
sST2	RV GCS	−0.04	−0.08, 0.01	0.10
	RV GLS	0.00	−0.04, 0.04	0.93
	LV GCS	0.03	−0.02, 0.08	0.27
	LV GLS	0.01	−0.04, 0.06	0.72
	RV EF	−0.01	−0.03, 0.01	0.56
	LV EF	−0.03	−0.05, −0.01	0.011
NTproBNP	RV GCS	0.11	0.00, 0.22	0.049
	RV GLS	−0.02	−0.12, 0.09	0.77
	LV GCS	−0.07	−0.20, 0.05	0.22
	LV GLS	−0.07	−0.20, 0.06	0.28
	RV EF	−0.02	−0.08, 0.03	0.38
	LV EF	0.02	−0.03, 0.07	0.45

*LV* left ventricle, *RV* right ventricle, *EF* ejection fraction, *GLS* global longitudinal shortening, *GCS* global circumferential shortening

**Table 3:** Adjusted models* of biomarkers on ventricular size and functional parameters

Outcome	Characteristic	Beta	95% CI	*p*-value
MMP1	RV GCS	0.07	0.00, 0.14	0.034
	Age at CMR	0.00	−0.02, 0.03	0.93
	Male	0.25	−0.15, 0.64	0.21
	RV EF	−0.04	−0.08, −0.01	0.014
	Age at CMR	0.00	−0.02, 0.03	0.78
	Male	0.11	−0.30, 0.52	0.60
sST2	RV GCS	−0.04	−0.08, −0.01	0.1
	Age at CMR	0.01	0.00, 0.03	0.11
	Male	0.24	0.00, 0.48	0.047
	LV EF	−0.02	−0.04, 0.00	0.050
	Age at CMR	0.01	0.00, 0.03	0.085
	Male	0.14	−0.11,0.39	0.26
NTproBNP	RV GCS	0.11	0.00,0.21	0.04
	Age at CMR	−0.02	−0.06, 0.01	0.21
	Male	−0.67	−1.3, −0.07	0.029
	RVEDV	0.005	−0.0046, 0.015	0.301

*Multivariable regression models include patient age at time of CMR and sex as covariates

*LV* left ventricle, *RV* right ventricle, *EF* ejection fraction, *GLS* global longitudinal shortening, *GCS* global circumferential shortening

## Discussion

This study evaluated cross-sectional associations between circulating biomarkers thought to represent myocardial remodeling and RV and LV function including strain as measured by CMR in patients with rTOF. We identified associations between MMP1 and RV dilation and RVGCS; between NTproBNP and RVGCS, and between ST2 and LVEF. We showed that some biomarkers varied by sex.

MMP1 has previously been shown to be associated with increased end-diastolic volumes and decreased ejection fraction in heart failure and hypertensive patients [32]. Similarly, in our study, increased MMP1 levels were associated with more RV dilation and worse ejection fraction. DiLorenzo et al. previously demonstrated an association between MMP1 and CMR parameters of ventricular remodeling (indexed RV and LV mass, RV mass to volume ratio) in this cohort [17]. Our study found an additional association of MMP1 levels with worse RV circumferential strain, but not with longitudinal strain. This association could be related to MMP1 activation in a fibrotic response during myocardial wall stress. In this scenario, MMP1 might be a marker for right ventricular dysfunction after TOF repair. However, whether circulating levels of MMP1 are directly related to myocardial extracellular matrix activation and collagen degradation cannot be proven in our study.

The association between MMP1 and RVGCS, but not RVGLS could be related to the unique myocardial architecture in TOF hearts, as shown by Sanchez-Quintana et al. in 1996 [33]. In an anatomically normal RV, the deep myofibers in the ventricle are arranged predominantly in a horizontal fashion and myocardial contraction is predominately longitudinal from the base of the heart to the apex [11]. In TOF, an additional layer of circumferential fibers is present in the RV similar to that seen in a normal LV [33]. Further, the superficial layer of the myocardium in TOF hearts has a more oblique orientation. Therefore, in the RV of TOF, there exists a predominance of circumferential contraction as compared to longitudinal contraction [34]. Thus, it stands to reason that MMP1 is associated with RVGCS, rather than RVGLS, in patients with TOF.

Prior studies have shown decreased RVGLS in TOF and that circumferential shortening is likely to offer compensatory shortening to the right ventricle with impaired longitudinal shortening [34–36]. We and others have studied longitudinal shortening of the RV and demonstrated this phenomenon with reduced tricuspid plane systolic excursion (TAPSE) [37, 38]. Thus, it might be more appropriate to use RVGCS, rather than RVGLS as the preferred measure of systolic functional assessment for patients with repaired TOF. However, additional longitudinal studies are needed to validate this hypothesis.

sST2 is released by the myocardium in response to wall stress and has previously been noted to be elevated in LV heart failure [39]. In our study, elevated sST2 was associated with worse LVEF consistent with prior publications. sST2 has also been shown to be associated with RV volume overload and progression of RV failure, although we did not identify associations with RV dilation [39, 40]. In adults, sST2 levels have been identified as having prognostic value in heart failure patients [41]. Some studies have shown a similar prognostic ability of sST2 levels in pediatric patients with dilated cardiomyopathy [41]. These findings suggest that in the pediatric population, sST2 might be useful for cardiac disease involving the left ventricle, rather than the right.

NTproBNP is a familiar biomarker which is secreted by the heart in response to a cardiac volume load and is often elevated in heart failure patients [39, 42]. In rTOF, NTproBNP has been correlated with RV dilation, severity of pulmonary valve regurgitation and exercise capacity [39]. In a study by Karali et al., associations between NTproBNP and RV fibrosis as measured by late gadolinium enhancement by CMR were seen [43]. To our knowledge, however, no significant associations between NTproBNP and RV strain by CMR have yet been identified. In our study, elevated NTproBNP was associated with abnormal RVGCS, independently of RV dilation. Thus, it is possible that NTproBNP could serve as a marker of RV dysfunction in the presence of volume load on the RV. When adjusting for gender, we found that NTproBNP levels were more elevated in females. Gender differences in NTproBNP levels in adults have been reported in the literature and future studies should adjust for gender [44, 45].

In the small group of patients with a history of PVR, we found more dilated ventricles. It is possible these patients may not have experienced reverse RV volume remodeling after PVR and thus remain more dilated. In future studies, it will be important to evaluate the time to RVEDV normalization, if at all, after PVR, and whether circulating biomarkers prior to PVR are associated with reverse remodeling of the RV.

In summary, MMP1 is a potential biomarker of RV volume and function (EF and strain), NTproBNP may serve as a biomarker for RV function measured as strain, while ST2 is potentially correlated with LV function. These biomarkers could potentially provide a way to identify patients at risk for RV dysfunction early on in rTOF and thereby guide timelier intervention. However, larger longitudinal studies are needed for validation.

## Limitations

This single-center study is limited by the small sample size. Due to the cross-sectional nature of the study, it lacks longitudinal data and therefore changes over time in biomarker levels, strain and functional parameters are not demonstrated. Given our relatively young patient population, the results may not be generalizable to adult patients with rTOF. Similarly, we cannot comment on age at PVR and the relation to strain due to the small and relatively young patient population studied. Additionally, as we evaluated multiple variables, there is the inherent potential of chance findings. Since the study measurements were obtained by a single observer with excellent intra-observer reliability, we did not provide inter-observer reproducibility, which should be considered in future, multicenter studies.

We performed CMR strain analyses using cvi42 exclusively and cannot generalize the results to other analytic software as inter-vendor differences may exist. The RV free wall is generally thin and can be difficult to accurately assess for myocardial deformation by CMR, thus potentially limiting our interpretation of RV strain parameters by CMR. Similarly, the presence of RVOT intervention may affect contouring for measurement of RV strain as might the presence of sternal wires for assessment of strain and EF. However, previous studies have shown that RV strain can be replicated with good intra- and inter-observer agreement [46]. Additionally, normative reference values with large validation studies are lacking for strain parameters by CMR [13, 47]. Thus, it may be more useful to trend strain by CMR in patients to evaluate for relative changes over time.

## Conclusion

In patients after TOF repair, biomarkers are associated with known measures of RV remodeling and LV function. Of the biomarkers tested, MMP1 and NTproBNP were associated with measures of RV remodeling, while sST2 was associated with measures of LV function. Additionally, assessing RV function with global circumferential strain might be of interest over global longitudinal strain given the RV adaptation to the disease itself and to residual lesions. Future analyses should adjust for patient sex. Longitudinal studies are necessary to validate these findings and to determine if biomarkers in conjunction with strain can preemptively identify patients at risk for poor outcomes after TOF repair.

## Data Availability

No datasets were generated or analysed during the current study.
